# RIG-I-like receptor activation by dengue virus drives follicular T helper cell formation and antibody production

**DOI:** 10.1371/journal.ppat.1006738

**Published:** 2017-11-29

**Authors:** Joris K. Sprokholt, Tanja M. Kaptein, John L. van Hamme, Ronald J. Overmars, Sonja I. Gringhuis, Teunis B. H. Geijtenbeek

**Affiliations:** 1 Department of Experimental Immunology, Academic Medical Center, University of Amsterdam, Amsterdam, the Netherlands; 2 Amsterdam Infection & Immunity Institute, Amsterdam, the Netherlands; Washington University, UNITED STATES

## Abstract

Follicular T helper cells (T_FH_) are fundamental in orchestrating effective antibody-mediated responses critical for immunity against viral infections and effective vaccines. However, it is unclear how virus infection leads to T_FH_ induction. We here show that dengue virus (DENV) infection of human dendritic cells (DCs) drives T_FH_ formation via crosstalk of RIG-I-like receptor (RLR) RIG-I and MDA5 with type I Interferon (IFN) signaling. DENV infection leads to RLR-dependent IKKε activation, which phosphorylates IFNα/β receptor-induced STAT1 to drive IL-27 production via the transcriptional complex ISGF3. Inhibiting RLR activation as well as neutralizing antibodies against IL-27 prevented T_FH_ formation. DENV-induced CXCR5^+^PD-1^+^Bcl-6^+^ T_FH_ cells secreted IL-21 and activated B cells to produce IgM and IgG. Notably, RLR activation by synthetic ligands also induced IL-27 secretion and T_FH_ polarization. These results identify an innate mechanism by which antibodies develop during viral disease and identify RLR ligands as potent adjuvants for T_FH_-promoting vaccination strategies.

## Author summary

Strong antibody production is critical for effective immune responses against viral infections and is a primary factor in the development of successful vaccines. However, it is unclear how virus infection leads to effective antibody responses. Dengue virus (DENV) is known to induce potent antibody production, although the underlying mechanism is poorly understood. Dendritic cells (DCs) are professional sentinels of the immune system and crucial for induction of immune responses. Here we show that DENV infection of human DCs leads to robust antibody production by inducing a specific T helper cell type (also called follicular T helper or T_FH_) that specializes in stimulating antibody production by B cells. Our data show that DENV replication triggers a viral detection system consisting of sensors RIG-I and MDA5, which specifically induce factors such as IL-27 that are essential for T_FH_ induction. Our data demonstrate that this viral detection system is especially powerful to induce antibody production. Indeed, synthetic molecules that trigger this viral detection mechanism induced superior antibody production compared to other activation signals. Thus, we have identified a viral detection mechanism that leads to strong antibody production and its importance in DENV infection as well as its potential in vaccinations.

## Introduction

Dengue virus is a global mosquito-transmitted pathogen that infects 400 mln people annually [1]. The majority of patients experience only mild fever, but the disease can progress to life-threatening dengue shock syndrome and dengue hemorrhagic fever. With no specific antivirals or effective vaccine available there is urgent need to advance our understanding of the human immune response against DENV to improve vaccine development and identify molecular targets for drug development.

Antibodies are critical for the host immune response to control, eradicate and prevent (future) viral infections. Antibodies play a dual role in DENV pathology as neutralizing high-affinity antibodies are protective while cross-reacting antibodies can possibly enhance disease of heterologous DENV strains via antibody-dependent enhancement of infection [2]. However, it is unclear how antibodies are induced upon DENV infection. High-affinity antibodies are formed by B cells in germinal centers (GC) during somatic hypermutation [3]. T_FH_ cells are critical for the formation and maintenance of GCs, and induce B cell proliferation and Ig isotype class switching by producing IL-21. T_FH_ cells selectively stimulate high-affinity B cell entry into GCs to promote effective antibody-mediated responses [3–5]. Formation of T_FH_ cells is driven by transcription factor Bcl-6, which induces IL-21 production and expression of chemokine receptor CXCR5, which is pivotal for T_FH_ migration into GCs [6]. DCs are essential for T_FH_ differentiation from naïve CD4^+^ T cells and previously we have shown that in humans IL-27 is pivotal for T_FH_ formation while IL-6 enhances T_FH_ formation in response to fucosylated parasitic/bacterial ligands [7].

DCs are equipped with numerous sensors including Toll-like receptors (TLRs) and RLRs that sense viral particles or viral replication products to induce innate signaling and drive T_H_ polarization for tailored adaptive immune responses. DENV can both activate TLRs as well as RLRs, depending on the cell type, leading to cytokine secretion and type I IFN production [8–10]. TLR3 resides in endosomes while RLRs are cytoplasmic receptors. Both their activation by viral RNA leads to IFN-β induction via Tank binding protein 1 (TBK1) and transcription factor IRF3. However, RLR signaling also involves IkB kinase (IKK)-related kinase IKKε, which functions in concert with TBK1 to activate IRF3 [11,12]. In parallel with IRF3-dependent IFN-β transcription, TLR3 and RLRs activate NFκB signaling to induce cytokine expression and the combined effect of type I IFN and cytokines determines which differentiation program is initiated in naïve CD4^+^ T cells. However, it remains unclear how viral sensing in DCs leads to T_FH_ development and subsequent B cell activation.

Here we show that DENV-infected DCs instruct naïve CD4^+^ T cells to differentiate into Bcl-6^+^CXCR5^+^PD-1^+^ IL-21-secreting T_FH_ cells. DENV RNA replication in both monocyte-derived and primary skin DCs triggers RLR RIG-I and MDA5 leading to IFN-β transcription and IFN-α/βR activation. Notably, RLR-induced IKKε activation modulates IFNα/βR signaling by phosphorylating STAT1. This results in the formation of transcriptional complex ISGF3 instead of STAT1 homodimers, which is pivotal for IL-27 production by DCs and T_FH_ formation. Inhibiting RLR signaling by silencing adapter protein MAVS abrogates IL-27 production and T_FH_ polarization by DENV-infected DCs. In addition, direct RLR activation by synthetic ligands is sufficient to induce IL-27 transcription and T_FH_ formation.

## Results

### DENV infection of DCs leads to T_FH_ polarization

**Fig 1:**
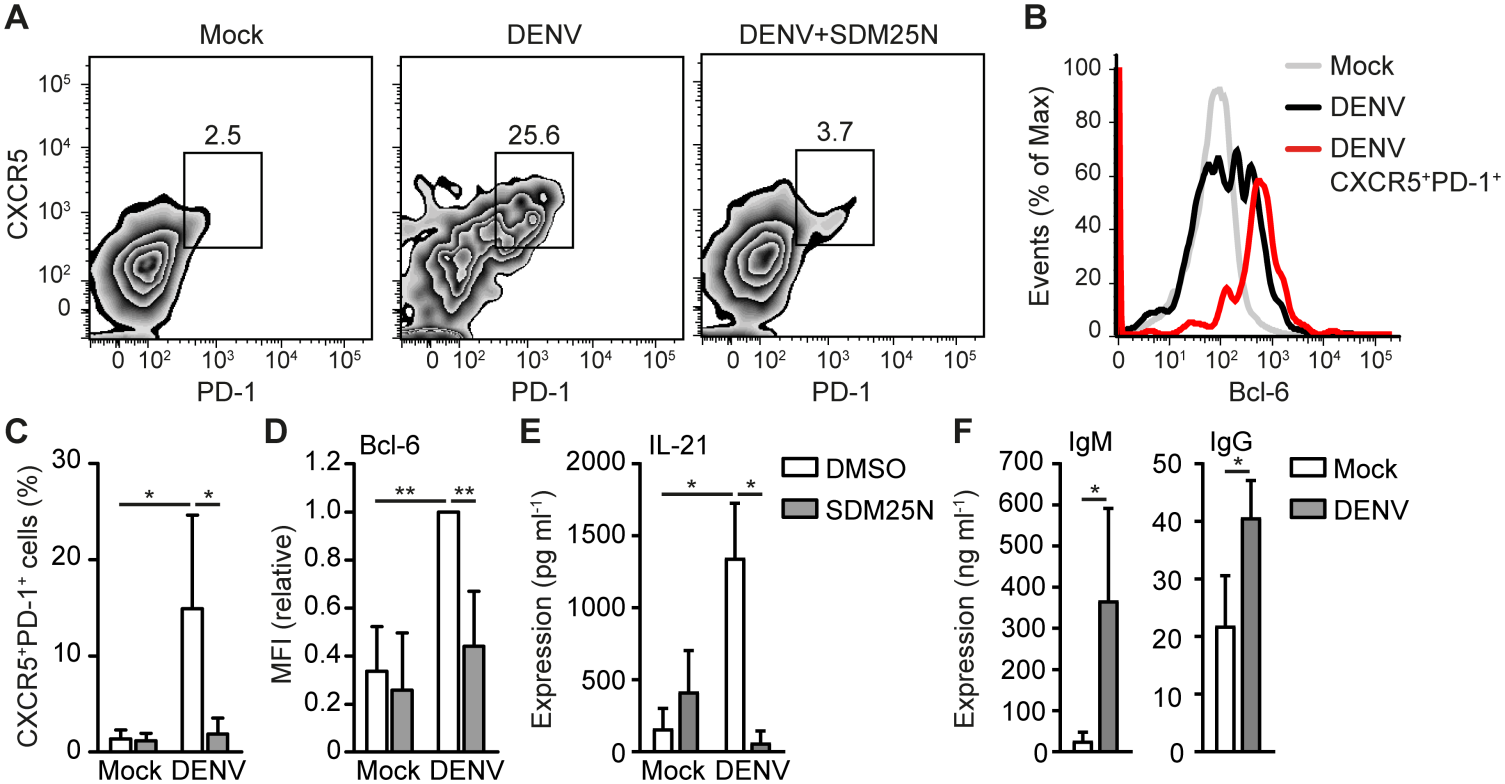
DENV infection of DCs induces Bcl-6^+^CXCR5^+^PD-1^+^ T_FH_ formation. Flow cytometry analysis of extracellular CXCR5, PD-1 (A,C) and intracellular Bcl-6 (B,D) expression of differentiated T cells after coculture of naive CD4+ T cells with mock-treated DC or DCs infected with DENV for 48h in the absence or presence of DENV replication inhibitor SDM25N. Numbers in zebra plots of (A) indicate percentage of gated cells. Histograms in (B) represent all T cells from mock coculture (grey), DENV coculture (black) or CXCR5^+^PD-1^+^ T cells from DENV coculture (red) as gated in (A). Results in (D) are relative to fluorescent intensity of DENV samples set as 1. (E) IL-21 in supernatant of differentiated T cells as described in (A) was measured by ELISA. (F) IgM and IgG in the supernatant of B cells cocultured for 7 days with differentiated T cells from mock-treated or DENV-infected DC-T cell cocultures was analyzed by ELISA. Data are representative of at least five (A) or four (B) independent experiments with different donors or are collated data (mean ± s.d.) of five (C), four (D), three (F) or two (E) different donors. ** *P*<0.01, * *P*<0.05 (student’s t-test). MFI: mean fluorescent intensity.

DCs drive T_H_ differentiation and therefore we examined DC-induced immune responses upon DENV infection. DENV efficiently infected human DCs and induced DC maturation as indicated by increased surface expression of CD83 and CD86 (S1 Fig). To investigate T_FH_ differentiation, DENV-infected DCs were co-cultured with naïve CD4^+^ T cells and T_FH_ induction was determined by measuring expression of CXCR5 and PD-1, which are both expressed by lymph node T_FH_ cells *in vivo* [13,14]. Strikingly, DENV-infection of DCs induced a robust CXCR5^+^PD-1^+^ subset of differentiated T_H_ cells (Fig 1A and 1C), which expressed high levels of T_FH_-specific transcription factor Bcl-6 (Fig 1B and 1D). T cell differentiation induced by DENV-infected DCs also resulted in strong secretion of IL-21, which is the main effector cytokine of T_FH_ cells (Fig 1E). To investigate whether DENV-induced T_FH_ cells have the capacity to activate B cells, we co-cultured DENV-differentiated T_H_ cells with CD19^+^ B cells and measured antibody production. Remarkably, differentiated T_H_ cells from DENV-infected, but not mock-treated DCs, induced secretion of both IgM and IgG by B cells (Fig 1F). Blocking DENV RNA replication and infection of DCs (S2 Fig) with DENV RNA replication inhibitor SDM25N [15] abolished the formation of IL-21-secreting CXCR5^+^PD-1^+^Bcl-6^+^ T_FH_ cells (Fig 1A and 1C–1E). These data strongly indicate that DENV replication in DCs induces a T_H_ differentiation program leading to T_FH_ induction and B cell activation.

### DENV RNA replication induces type I IFN responses

**Fig 2:**
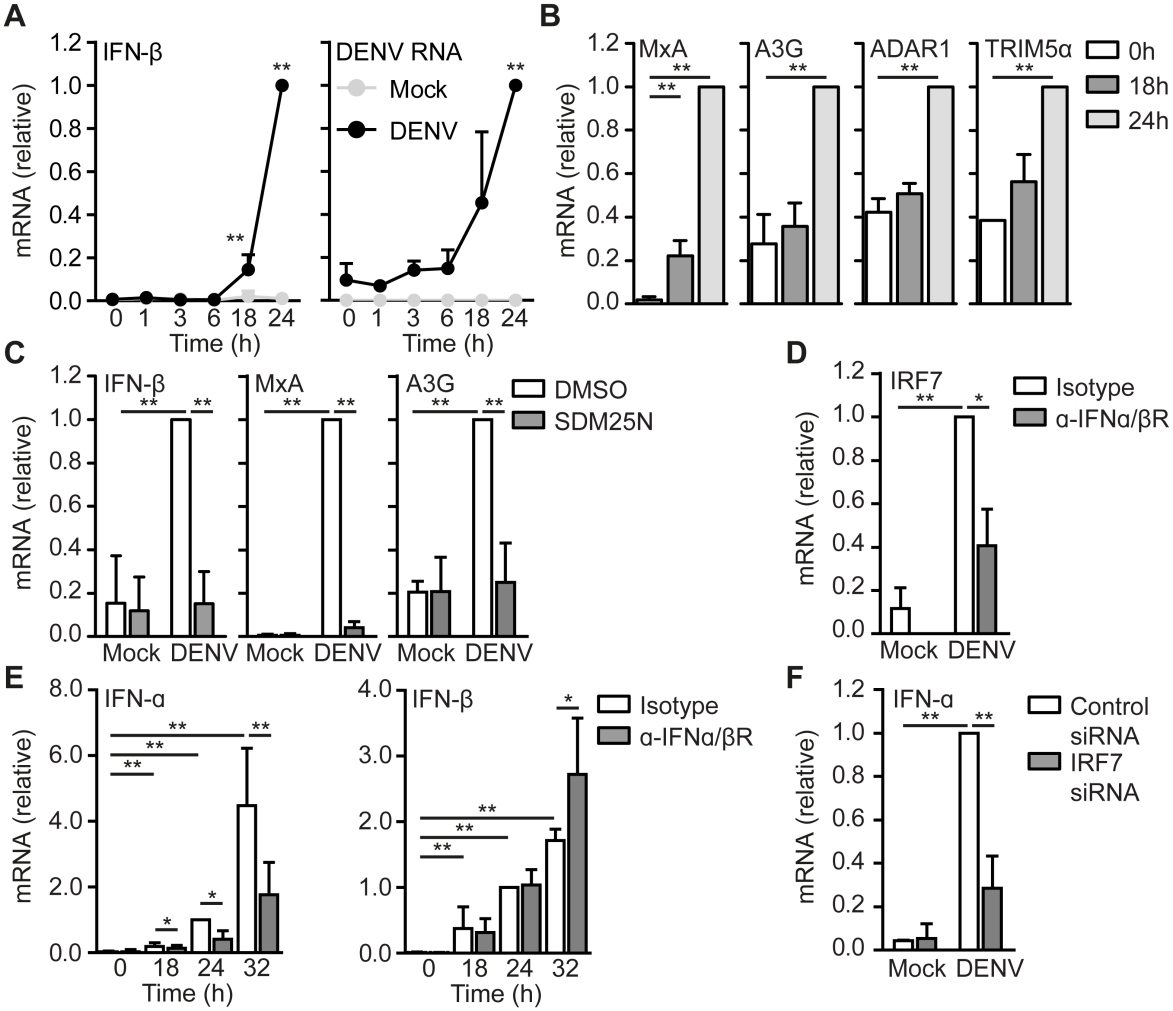
DENV RNA replication induces type I IFN responses. IFN-β, DENV RNA, MxA, APOBEC3G, ADAR1 or TRIM5α mRNA expression in mock-infected or DENV-infected DCs over time (A,B) was measured by real-time PCR, normalized to GAPDH and set as 1 in DENV-infected DCs. (C) mRNA expression was measured similar as in (A) but in the presence or absence of SDM25N 24 hours post infection (h.p.i.). (D,E) mRNA expression was measured similar as in (A) but in the presence or absence of α-IFNα/βR 24 h.p.i. (D) or over time (E). mRNA expression of DENV-infected samples treated with isotype control of 24h were set at 1. (F) mRNA expression was measured similar as in (A) but after transfecting cells with IRF7 or control siRNA. Data are collated (mean ± s.d.) of at least five (E), three (A,B,C,F two for TRIM5α) or two (D) different donors. ** *P*<0.01, * *P*<0.05 (student’s t-test). A3G, APOBEC3G.

We set out to identify the molecular mechanism in DCs essential for T_FH_ differentiation. We investigated induction of type I IFN responses upon DENV infection. DENV induced IFN-β transcription in DCs at 18 hours post infection (h.p.i.), which increased over time and correlated with DENV RNA replication (Fig 2A). Next we measured induction of antiviral IFN stimulated genes (ISGs), which are induced by IFNα/βR signaling and indicative of functional type I IFN responses [16]. DENV-infection of DCs induced expression of ISGs MxA, APOBEC3G, ADAR1 and TRIM5α 24 h.p.i. (Fig 2B). These responses depended on DENV RNA replication as the inhibitor SDM25N abrogated the induction of IFN-β and ISGs (Fig 2C). IRF7 is crucial for the induction of IFN-α, which is required for enhancing type I IFN responses [17]. Interestingly, DENV infection induced IRF7 and IFN-α expression after IFN-β induction (Fig 2D and 2E). Both IRF7 and IFN-α expression depended on IFNα/βR signaling, while IFN-β expression was not decreased by blocking IFNα/βR antibodies at early time points (Fig 2D and 2E). IFN-β increased at 32 h.p.i. upon blocking IFNα/βR, probably because of increased DENV replication due to absence of antiviral ISGs (Fig 2E). Notably, silencing IRF7 using RNA interference abrogated IFN-α expression (Fig 2F). Thus, DENV RNA replication induces functional type I IFN responses that are initiated by IFN-β. The induction of IFNα/βR-dependent IRF7 expression subsequently drives IFN-α expression to increase and prolong type I IFN responses against DENV.

### DENV RNA replication activates RIG-I and MDA5

**Fig 3:**
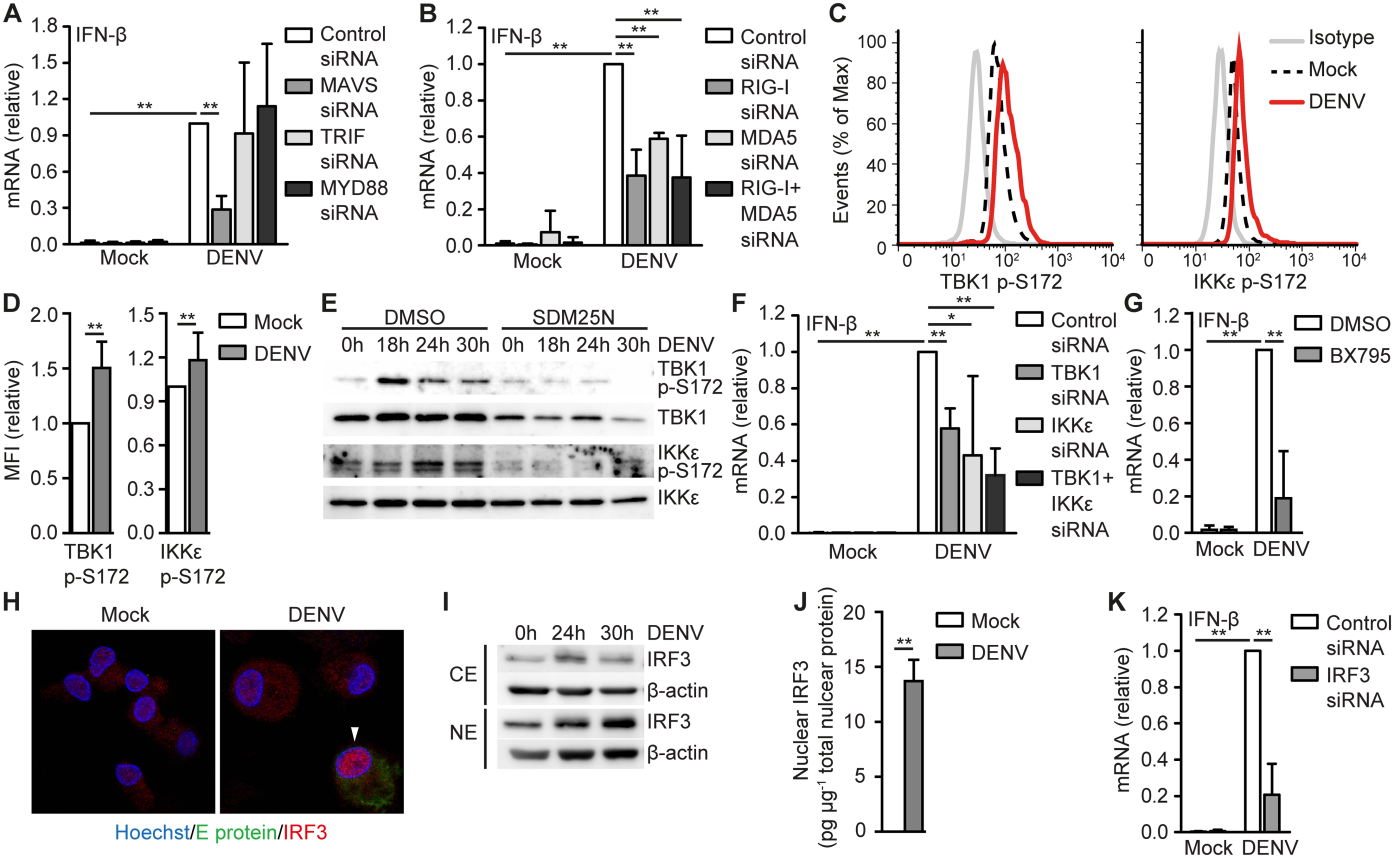
DENV RNA replication triggers RIG-I and MDA5. IFN-β mRNA expression 24 hours post infection (h.p.i.) of DCs that were infected with DENV (A,B,F,K) after MAVS, TRIF, MYD88 (A), RIG-I, MDA5 (B), TBK1, IKKε (F) or IRF3 (K) silencing by RNA interference was measured by real-time PCR, normalized to GAPDH and set as 1 in DENV-infected DCs treated with control siRNA. (C,D) Flow cytometry analyses of TBK1 or IKKε phosphorylation at Ser172 in mock-treated or DENV-infected DCs 14 h.p.i. using phospho-specific antibodies. Data in (D) show relative mean fluorescent intensity (MFI). (E) Immunoblot of whole cell lysates of DCs mock-treated or infected with DENV in the presence or absence of DENV replication inhibitor SDM25N for TBK1 p-S172 and IKKε p-S172. Total TBK1 or IKKε served as loading control. (G) IFN-β mRNA of DENV-infected DCs 24 h.p.i. in the presence or absence of TBK1/IKKε inhibitor BX795 was measured by real-time PCR, normalized to GAPDH and set as 1 in DENV-infected DCs. (H) Confocal imaging of DNA (Hoechst, blue), DENV Envelope protein (E protein, green) or IRF3 (red) in mock-treated or DENV-infected DCs 18 h.p.i. White arrowheads indicate cells with nuclear IRF3. (I) Immunoblot for IRF3 of nuclear (NE) and cytoplasmic extracts (CE) of DCs infected with DENV at indicated times. β-actin served as loading control. (J) Similar as in (I) but IRF3 levels in nuclear extracts were measured by ELISA. Data are representative of at least five (H), four (C) or two (E,I) independent experiments with different donors or are collated (mean ± s.d.) of at least six (K), five (A) or four (B,D,F), three (G) or two (J) different donors. ** *P*<0.01, * *P*<0.05 (student’s t-test).

Both TLRs and RLRs have been implicated in DENV sensing [8–10]. To elucidate the PRR involved in DENV-induced IFN responses in DCs, we silenced adapter molecules TRIF/MYD88 and MAVS, which are essential for TLR and RLR signaling, respectively (S3 Fig). Silencing MAVS strongly decreased IFN-β and ISG expression in DENV-infected DCs, in contrast, neither silencing of TRIF nor MYD88 affected type I IFN responses (Fig 3A, S2 Fig) even though their silencing abrogated responses to known TLR ligands (S4 Fig). Notably, silencing RIG-I and MDA5 alone or together decreased DENV-induced IFN-β as well as ISG expression (Fig 3B, S2 Fig). RLR triggering leads to activation and phosphorylation of IKKε and TBK1, which target transcription factor IRF3 for nuclear translocation to drive IFN-β expression. DENV infection induced phosphorylation of TBK1 and IKKε, which was dependent on DENV RNA replication (Fig 3C, 3D and 3E). Silencing of TBK1 and IKKε or treatment with the TBK1/IKKε inhibitor BX795 strongly decreased DENV-induced IFN-β expression and supports an important role for these kinases in the type I IFN response against DENV (Fig 3F and 3G). Moreover, DENV infection resulted in nuclear translocation of IRF3 (Fig 3H, 3I and 3J) and silencing IRF3 abrogated IFN-β expression (Fig 3K). These data show that RIG-I and MDA5 sense DENV RNA replication and induce type I IFN responses via TBK1 and IKKε-mediated IRF3 signaling.

### RLR triggering suppresses DENV RNA replication via type I and type III IFN responses

**Fig 4:**
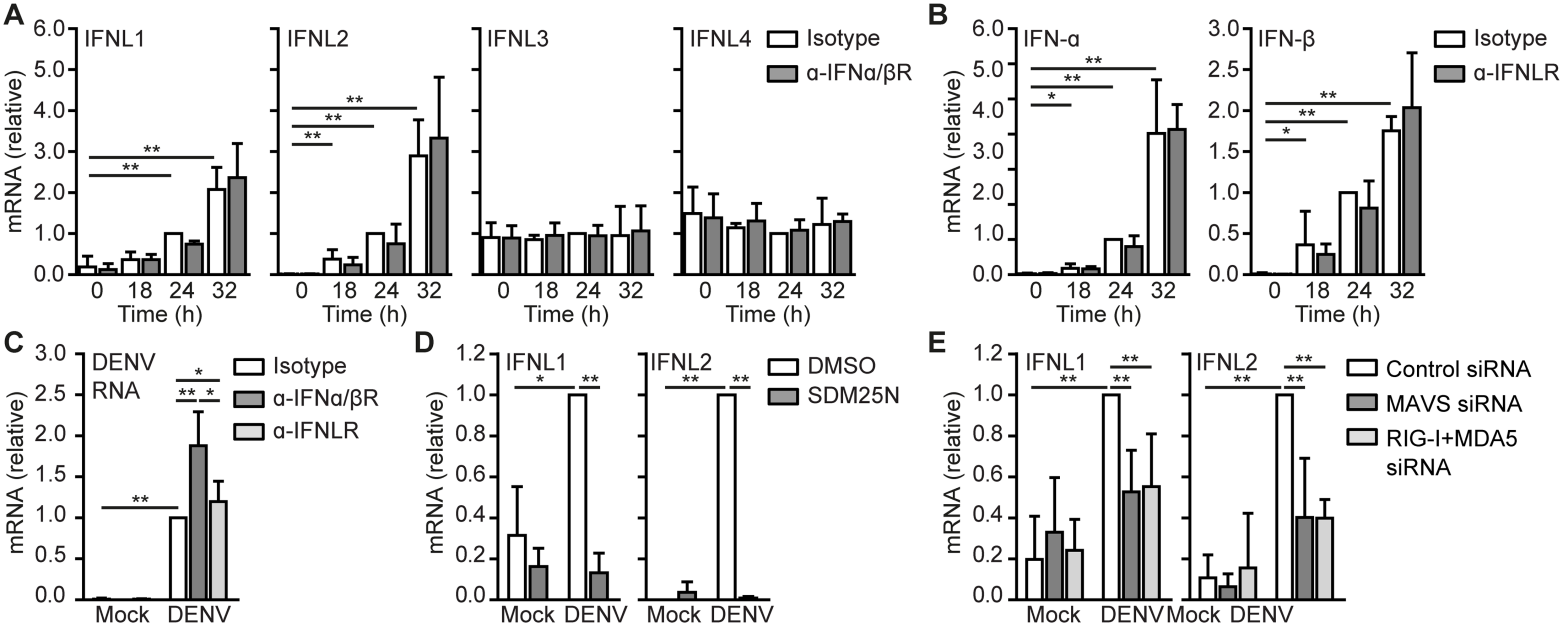
DENV-induced RLR triggering leads to antiviral type I IFN and type III IFN responses. IFNL1, IFNL2, IFNL3, IFNL4, IFN-α, IFN-β mRNA expression and DENV RNA expression in mock-infected or DENV-infected DCs over time in the presence or absence of blocking antibodies directed against IFNα/βR (A,C) or IFNLR (B,C) or in the presence or absence of DENV RNA replication inhibitor SDM25N (D) was measured by real-time PCR, normalized to GAPDH and set as 1 in DENV-infected DCs at 24 hours post infection. (E) Similar as in (A), but DCs were transfected with MAVS, RIG-I or MDA5 siRNA and IFNL1 and IFNL2 mRNA expression was analyzed 24h post infection. Data are collated (mean ± s.d.) of at least four (B,E), three (A,C) or two (D) different donors. ** *P*<0.01, * *P*<0.05 (student’s t-test).

Parallel to type I IFN responses, RIG-I and MDA5 induce type III IFN responses which have been implicated in DC migration, viral suppression and modulation of T and B cell responses [18–21]. Therefore, we investigated type III IFN responses by analyzing the expression of IFN-λ genes (IFNL1-4). DENV induced the expression of IFNL1 and IFNL2 and these responses were not affected by type I IFN as blocking IFNα/βR signaling did not affect IFNL1 or IFNL2 expression (Fig 4A). Blocking IFNLR did also not impact DENV-induced IFN-α or IFN-β responses indicating that type I and type III responses operate independently (Fig 4B). IFN-λ is known to suppress viral replication and therefore we examined the effect of blocking IFNLR antibodies on DENV RNA replication. Blocking IFNLR increased DENV RNA replication but not to a similar extent as blocking IFNα/βR (Fig 4C). These data suggest that although type I and type III IFN suppress DENV replication, type I IFN is more effective than type III IFN. Next, we investigated how IFN-λ is induced by DENV. Interestingly, the replication inhibitor SDM25N abrogated DENV-induced IFNL1 and IFNL2 expression (Fig 4D). Moreover, IFNL1 and IFNL2 were induced by MAVS, RIG-I and MDA5 as silencing MAVS or RIG-I and MDA5 together strongly decreased both IFNL1 and IFNL2 expression by DENV infection (Fig 4E). These data strongly suggest that RIG-I and MDA5 triggering by DENV replication induces type III IFN responses that operate independently of type I IFN to suppress viral replication.

### RLR activation by DENV induces IL-27 production

**Fig 5:**
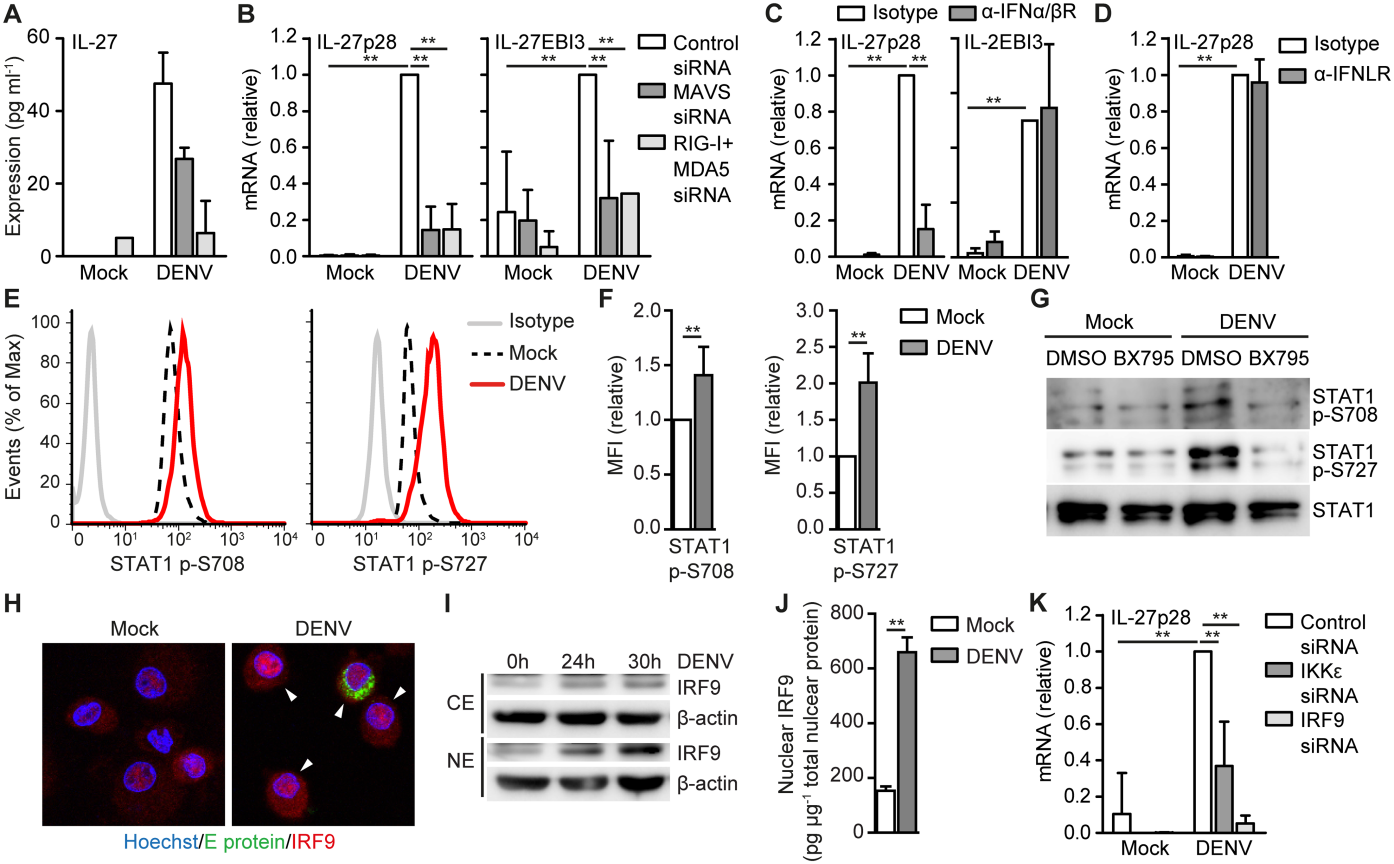
DENV-mediated RLR activation induces IL-27 via IFNα/βR signaling. DCs were mock-treated or infected with DENV for 30 (E,F,G,H,I), 32 (B,C,D,K) or 48 hours (A) in the presence or absence of blocking antibodies directed against IFNα/βR (C),IFNLR (D) or after MAVS, RIG-I, MDA5, IKKε, or IRF9 silencing (A,B,K) and secretion of IL-27 into the supernatant was measured by ELISA (A) or mRNA expression was analyzed using real-time PCR (B,C,D,K), normalized to GAPDH and set at 1 in DENV-infected samples treated with control siRNA (B,K) or isotype antibody (C,D). (E-G) STAT1 phosphorylation at Ser708 or Ser727 in mock-treated or DENV-infected DCs was analyzed by flow cytometry (E,F) or immunoblot (G) using phospho-specific antibodies. Total STAT1 served as loading control in (G). Relative mean fluorescent intensity (MFI) shown in (F). (H) Confocal imaging of DNA (Hoechst, blue), DENV Envelope protein (E protein, green) or IRF9 (red) in mock-treated or DENV-infected DCs 30 hours post infection. White arrowheads indicate cells with nuclear IRF9. (I) Immunoblot for IRF9 of nuclear (NE) or cytoplasmic (CE) extracts of DCs infected with DENV at indicated times. β-actin served as loading control. (J) Similar as in (I) but IRF9 levels in nuclear extracts were measured by ELISA. Data are representative of at least five (E), four (H), or two (A,G,I) independent experiments with different donors (mean ± s.d. of duplicates in A) or are collated data (mean ± s.d.) of four (D,F,K),three (B,C) or two (J) different donors. ** *P*<0.01, * *P*<0.05 (student’s t-test).

Next, we investigated induction of cytokines involved in T_FH_ differentiation. Mice lacking IL-27R have impaired T_FH_ formation [22] and we have recently shown that IL-27 is crucial for T_FH_ polarization by human DCs in response to fucosylated parasitic/bacterial ligands [23]. In addition, Activin A and IL-12 are known to be important factors to drive human T_FH_ formation [24,25]. Therefore, we examined whether DENV infection leads to Activin A, IL-12 or IL-27 expression. IL-12 is heterodimeric protein consisting of subunit p35 and p40. Although DENV induced low levels of IL-12p35, we were unable to detect IL-12p40 expression and this resulted in a lack of IL-12p70 protein (S5A and S5B Fig). We were also unable to detect increased Activin A expression in DENV-infected cells, while LPS strongly induced both IL-12p70 and Activin A (S5A and S5B Fig). Direct stimulation of RIG-I and MDA5 with RLR ligand poly(I:C)Lyovec also did not induce IL-12p70 production or Activin A expression (S5A and S5B Fig), indicating that these factors are more associated with TLR-induced T_FH_ formation and that RLR-mediated T_FH_ differentiation depends on other factors. Interestingly, DENV infection of DCs induced IL-27 production, which decreased by silencing either MAVS or both RIG-I and MDA5 (Fig 5A). IL-27 is a heterodimeric cytokine consisting of subunit p28 and Epstein-Barr virus-induced gene 3 (EBI3) [26]. DENV infection induced expression of both IL-27p28 and EBI3, which was dependent on RLR signaling as well as viral replication (Fig 5B; S5C Fig). Furthermore, blocking IFNα/βR antibodies abrogated IL-27p28 expression without affecting EBI3 expression (Fig 5C), supporting differential regulation of IL-27p28 and EBI3 [27,28] and an important role for crosstalk between RLR and IFNα/βR signaling. Interestingly, IL-27p28 expression was not affected by blocking IFNLR antibodies indicating that specific IFN signaling is necessary for IL-27p28 expression (Fig 5D).

Il-27p28 contains an IFN-stimulated response element (ISRE), which is induced by IFN-stimulating gene factor 3 (ISGF3), a complex of STAT1, STAT2 and IRF9 [27]. Differential signaling by IFNα/βR triggering leads to induction of either STAT1 homodimers or ISGF3 induction [29]. As ISGF3 formation is controlled by IKKε-dependent phosphorylation of STAT1 at Ser708, which prevents STAT1 homodimer formation [30] and IKKε also enhances the transactivation capacity of ISGF3 by phosphorylating STAT1 at Ser727 [23], we investigated whether ISGF3 was involved in IL-27 induction upon DENV infection. We observed STAT1 phosphorylation at Ser708 and Ser727 which was abrogated after treatment with TBK1/IKKε inhibitor BX795 (Fig 5E, 5F and 5G). We also observed nuclear translocation of IRF9 in DENV infected DCs (Fig 5H, 5I and 5J). Moreover, both IKKε and IRF9 were crucial for IL-27p28 expression since silencing of IKKε or IRF9 strongly decreased IL-27p28 expression (Fig 5K). These data show that RLR activation by DENV leads to IFN-β production and subsequent IFNα/βR triggering, and that IKKε activation modulates IFNα/βR signaling to drive IL-27 production.

### RLR signaling in DCs drives T_FH_ polarization

**Fig 6:**
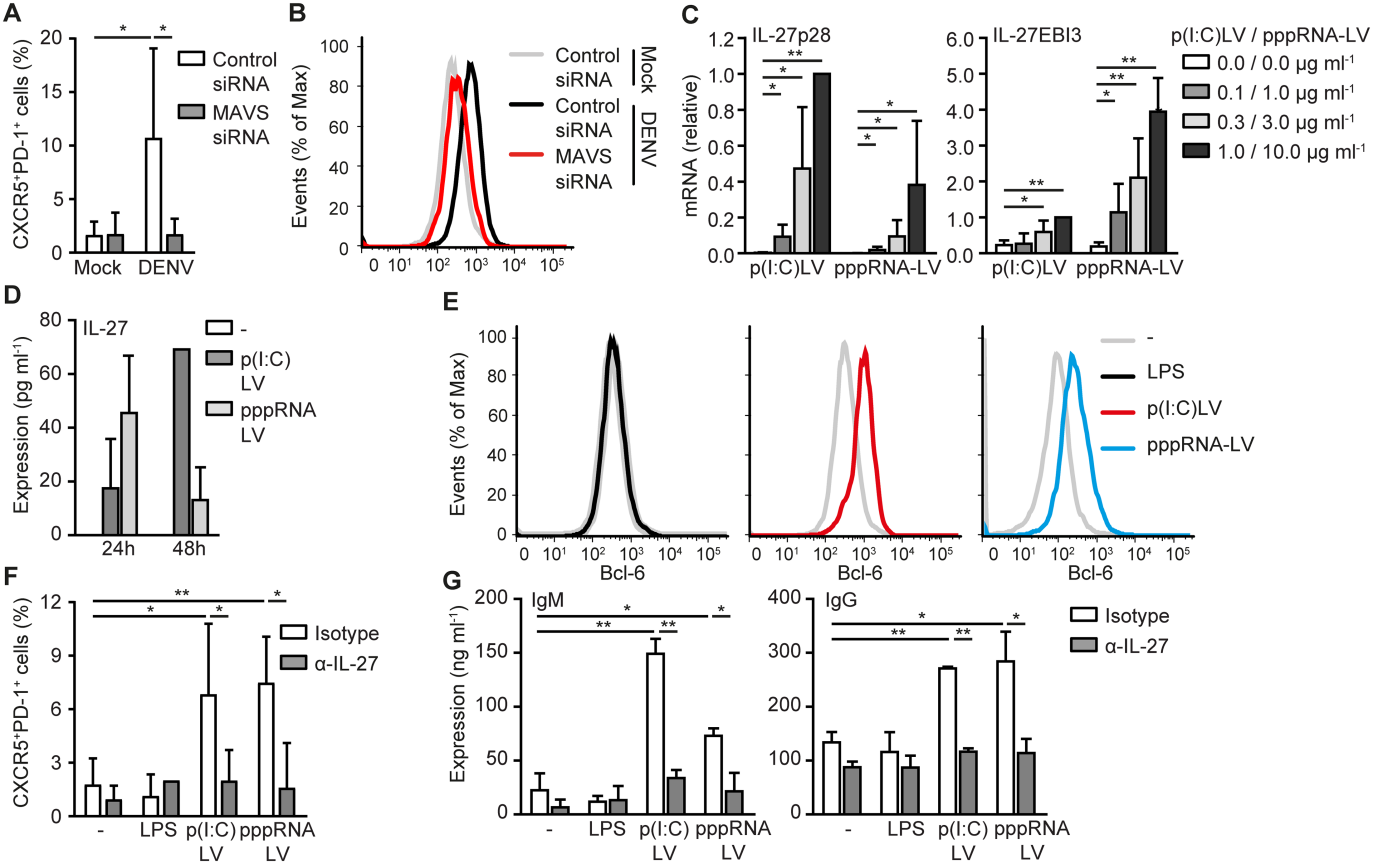
RLR activation by DENV or synthetic ligands drives T_FH_ formation via IL-27. Flow cytometry analysis of extracellular CXCR5, PD-1 (A,F) and intracellular Bcl-6 (B,E) expression of differentiated T cells resulting from cocultured naive CD4+ T cells with mock-treated DC or DCs infected with DENV (A,B) or stimulated with LPS, poly(I:C)LyoVec or 5’pppRNA-LyoVec (E,F) for 48h in the absence or presence of neutralizing antibodies against IL-27 (F) or after silencing MAVS (A,B). (C) IL-27p28 and IL-27EBI3 mRNA levels in DCs stimulated with poly(I:C)LyoVec or 5’pppRNA-LyoVec for 8h were analyzed using real-time PCR, normalized to GAPDH and set at 1 in 1.0 μg ml^-1^ stimulated poly(I:C)LyoVec samples. (D) IL-27 in the supernatant of untreated, poly(I:C)LyoVec or 5’pppRNA-LyoVec stimulated DCs after 24h or 48h was analyzed by ELISA. (G) IgM and IgG in the supernatant of B cells cocultured for 7 days with differentiated T cells from cocultures of naive CD4+ T cells with untreated, LPS, poly(I:C)LyoVec or 5’pppRNA-LyoVec stimulated DCs was analyzed by ELISA. Data are representative of at least three (D,E) or two (B) independent experiments with different donors (mean ± s.d. of duplicates in, D) or are collated data (mean ± s.d.) of three (G), four (C,F) or two (A) different donors. ** *P*<0.01, * *P*<0.05 (student’s t-test). -, unstimulated; p(I:C)LV, poly(I:C)LyoVec; pppRNA-LV, 5’pppRNA-LyoVec.

We next set out to investigate the importance of RLR sensing in T_FH_ formation by DENV-infected DCs. Silencing of MAVS abrogated the formation of CXCR5^+^PD-1^+^ T_FH_ cells by DENV-infected DCs (Fig 6A). Moreover, silencing of MAVS diminished Bcl-6 expression in T cells polarized by DENV-infected DCs (Fig 6B). These data strongly suggest that MAVS activation by DENV is pivotal for the formation of T_FH_ cells and identifies an important role for RLR activation in T_FH_ cells induction. To examine this, we transfected DCs with poly(I:C) or 5’pppRNA to activate RIG-I and MDA5 or RIG-I alone, respectively, and investigated T_FH_ differentiation. Strikingly, stimulation of DCs with either poly(I:C)Lyovec or 5’pppRNA-Lyovec induced CXCR5^+^PD-1^+^ T_FH_ formation and increased Bcl-6 expression (Fig 6E and 6F). Notably, T_H_ cells polarized by poly(I:C) or 5’pppRNA transfected DCs activated B cells to produce IgM and IgG (Fig 6G). In contrast, LPS-stimulated DCs did neither induce T_FH_ formation nor B cell activation (Fig 6E–6G). We next investigated the importance of IL-27 secretion by DCs for T_FH_ formation. Interestingly, both poly(I:C) and 5’pppRNA transfected DCs produced IL-27 (Fig 6C and 6D) and neutralizing antibodies against IL-27 abrogated CXCR5^+^PD-1^+^ T_FH_ formation (Fig 6F). Neutralizing IL-27 also diminished the capacity of T_H_ cells polarized by poly(I:C) or 5’pppRNA transfected DCs to activate B cells to produce IgM and IgG (Fig 6G). These data strongly indicate that RLR activation, either by DENV or synthetic ligands, drives IL-27-dependent T_FH_ polarization.

### CD14^+^ dermal DCs produce IL-27 in response to DENV RNA replication

**Fig 7:**
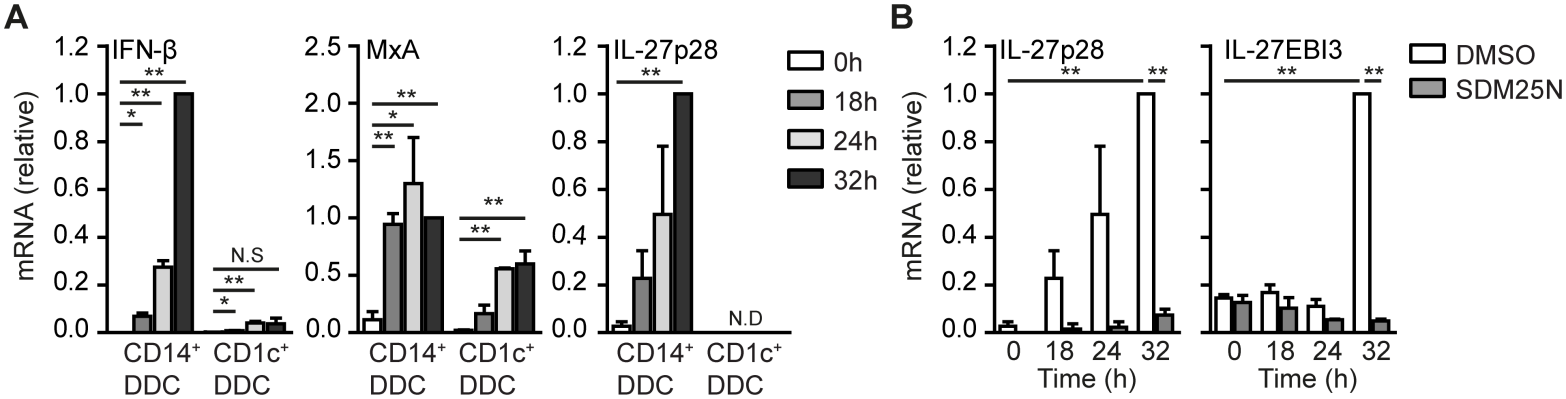
DENV RNA replication induces IL-27 expression in dermal CD14^+^ DCs. (A) CD14^+^ and CD1c^+^ dermal DCs were isolated from human skin and mock-treated or infected with DENV for indicated times before mRNA expression of IFN-β, MxA and IL-27p28 was analyzed using real-time PCR, normalized to GAPDH and set at 1 in 32h infected samples of CD14^+^ dermal DCs. (B) Similar to (A), but in the presence or absence of DENV replication inhibitor SDM25N. Data are collated (mean ± s.d.) of two independent experiments with different donors. ** *P*<0.01, * *P*<0.05 (student’s t-test). N.S., not significant; N.D. not detected.

DENV infection is initiated after a mosquito bite in the skin and swift immune responses against DENV depends on activation of skin DCs [31]. Human skin harbors several DC subsets including CD14^+^ and CD1c^+^ dermal DCs of which CD14^+^ dermal DCs are specialized in the induction of T_FH_ cells [32]. Therefore, we isolated CD14^+^ and CD1c^+^ dermal DCs from human skin and investigated if dermal DCs mount immune responses against DENV. Interestingly, DENV induced type I IFN responses in CD1c^+^ as well as CD14^+^ dermal DCs, although the induction of type I IFN was more robust in CD14^+^ dermal DCs than in CD1c^+^ dermal DCs (Fig 7A). Notably, CD14^+^ dermal DCs specifically expressed IL-27p28 in response to DENV infection (Fig 7A). We next set out to investigate the importance of DENV replication in IL-27p28 expression by CD14^+^ dermal DCs. Remarkably, DENV RNA replication inhibitor SDM25N abrogated the induction of IL-27p28 as well as IL-27EBI3 in DENV infected CD14^+^ dermal DCs (Fig 7B). These data indicate that human dermal DCs mount type I IFN responses against DENV and that DENV replication is essential to trigger IL-27 expression in CD14^+^ dermal DCs.

## Discussion

T_FH_ cells play a key role in antibody-mediated responses during viral infections or vaccination. Although numerous studies have demonstrated the importance of T_FH_ cells in GC reactions [3–5], little is known about the factors that drive T_FH_ differentiation from naïve CD4^+^ T cells upon viral infection. Here we show that DENV induces RLR-crosstalk with IFNα/βR signaling leading to IL-27 secretion, which is pivotal for the formation of IL-21-producing CXCR5^+^PD-1^+^Bcl-6^+^ T_FH_. DENV replication triggered RLR signaling leading to IKKε activation, which is essential for RLR-IFNα/βR crosstalk by phosphorylating STAT1 and inducing ISGF3 formation. Notably, direct activation of RLRs in DCs using poly(I:C)Lyovec or 5’pppRNA-Lyovec also induced IL-27 secretion, T_FH_ polarization and IgM and IgG production by B cells. These data strongly suggest that RLRs are efficient in the induction of T_FH_ responses via their crosstalk with IFNα/βR signaling, and links viral recognition to induction of robust antibody responses.

Viral RNA is a potent pattern-associated molecular pattern that can activate numerous receptors and induce strong immune responses; both TLRs and RLRs have been implicated in DENV sensing [8–10]. DENV particles contain positive single-stranded RNA that can be directly sensed by TLR7 as shown in macrophages [8]. However, we did not find a role for TLR7 in recognizing DENV by DCs as MYD88 silencing did not affect type I IFN responses, probably because TLR7 signaling requires IRF7, which is constituently expressed in plasmacytoid DCs but minimally in other DC subsets [17,33]. Indeed, our data show that IRF7 is minimally expressed by DCS and that this transcription factor is induced by DENV infection of DCs via IFN-β. DENV replication leads to double stranded RNA intermediates, which can be sensed by TLR3 and RLRs. Studies in multiple cell lines have shown that TLR3 can be involved in IFN-β production in response to DENV infection although it is unclear how cytoplasmic RNA is transferred to endosomal TLR3 [9,34]. Our data strongly suggest that RIG-I and MDA-5 but not TLR3 are involved in sensing of DENV and subsequent induction of type I and type III IFN responses. Although DENV is known to block type I IFN responses by inhibiting RLR-MAVS interaction, TBK1 and IRF3 phosphorylation and IFNα/βR signaling via STAT2 degradation [35–38], our data suggest that the inhibition does not effectively occur in DCs. Both type I IFN and type III IFN suppressed viral replication of DENV and preventing antiviral ISG induction by IFNα/βR or IFNLR increased DENV RNA replication. Notably, the increase in DENV RNA replication resulted in an increase in IFN-β suggesting that DENV RNA and IFN-β levels are correlated. It is likely that sensing of RNA products precedes activity of de novo produced viral proteins that block RLR signaling.

Recently, it was shown that Measles virus (MV) directly affects RLR activation. RIG-I and MDA5 activation is tightly regulated and requires dephosphorylation by PP1 phosphates for activation [39]. MV replication is sensed by RLRs and leads to type I IFN responses. However, MV triggers C-type lectin receptor DC-SIGN signaling leading to kinase Raf-1 activation [23]. Raf-1 subsequently induces association of inhibitor protein I-1 with PP1 to lower RIG-I and MDA5 dephosphorylation and type I IFN induction [40]. Although DENV also binds to DC-SIGN [41,42], we did not observe inhibition of RLR activation upon infection. In contrast to MV infection, which activates RLRs very rapidly [43], DENV infection is only sensed after 18 hours when innate signaling by DC-SIGN is probably not effective anymore. Therefore, RIG-I and MDA5 activation is not only important for T_FH_ polarization and antibody production but also to limit viral replication in DCs and possible viral transmission to other cells. Indeed, our data show that RLR-dependent induction of type I IFN and type III IFN suppressed DENV.

We have recently shown that IL-27 is important in the differentiation of T_FH_ and IL-27 induction is dependent on the formation of ISGF3 [7]. Although several TLRs can induce IL-27 transcription, the levels are not sufficient to induce T_FH_ polarization and requires IKKε activation by other receptors for STAT1 phosphorylation and ISGF3 formation [7]. RLRs are therefore unique in their ability to activate both IKKε and IFNα/βR signaling for efficient IL-27 transcription and T_FH_ polarization. These underlying mechanisms might also apply to other viruses as Measles virus, Influenza virus, Rubella virus, HIV-1 and Hepatitis C virus activate RLRs during infection [44–47]. Indeed, our data strongly suggest the use of RLR-ligands as adjuvants for human vaccination strategies, which has been shown to be successful in animal models [48–50].

In addition to IL-27, Activin A and IL-12 have been identified as important cytokines to drive human T_FH_ differentiation [24]. IL-12 expression is strongly induced by TLR signaling, while it is inhibited by RIG-I mediated IRF3 activation [51]. Our data also show that RLR triggering by synthetic ligands or DENV does not lead to IL-12p70 production, in contrast to strong IL-12p70 production by TLR4 activation. We obtained similar results for Activin A, suggesting that Activin A and IL-12p70 could be important for TLR-mediated T_FH_ formation while IL-27 is crucial for RLR-mediated T_FH_ formation.

In the natural course of infection, skin DCs are the first immune cells to encounter DENV after a blood meal of an infected mosquito [31,52]. Effective control of viral propagation from the site of infection requires robust type I IFN responses to suppress viral replication. Our data show that both CD14^+^ and CD1c^+^ dermal DCs mount type I IFN responses against DENV. Interestingly, DENV specifically induced IL-27 expression in CD14^+^ dermal DCs, which are known to be effective inducers of T_FH_ differentiation [32]. Our data show that the induction of IL-27 by DENV in CD14^+^ dermal DCs critically depends on DENV RNA replication and thereby supports a key function of cytoplasmic sensors of DENV RNA replication in the induction of T_FH_ responses by primary human skin DCs in response to DENV infection.

Our data shows that IFN-α/β induced IL-27 expression is pivotal for T_FH_ formation by DCs while direct stimulation of naïve CD4^+^ cells with type I IFN-α/β is thought to inhibit T_FH_ formation [24,53]. These studies indicate that IFN signaling, depending on the cell-type and time, can have different effects on T_FH_ differentiation. In addition, direct IFN-α/β stimulation does not lead to IL-27 transcription without IKKε activation to modulate IFNα/βR signaling.

Developing effective DENV vaccines has been hampered by the formation of non-neutralizing antibodies that have the potential to enhance disease [2,54]. A subunit vaccine based on the neutralizing epitope of DENV envelop protein could circumvent the formation of non-neutralizing antibodies [55,56]. However, subunit vaccines usually have low immunogenicity and induce only weak antibody responses. We propose that a subunit vaccine containing the neutralizing DENV epitope in combination with RLR-based adjuvants is a potent strategy to induce high levels of DENV neutralizing antibodies.

In conclusion, we have identified an innate mechanism in DCs that drives T_FH_ polarization during viral infection. Adjuvants targeting this innate mechanism have the potential to improve vaccination strategies for DENV and other pathogens.

## Materials and methods

### Ethics statement

This study was done in accordance with the ethical guidelines of the Academic Medical Center and human material was obtained in accordance with the AMC Medical Ethics Review Committee (i.e. Institutional Review Committee) according to the Medical Research Involving Human Subjects Act. Buffy coats obtained after blood donation (Sanquin) or skin tissue are not subjected to informed consent according to the Medical Research Involving Human Subjects Act and the AMC Medical Ethics Review Committee. All samples were handled anonymously.

### DC stimulation and RNA interference

Peripheral blood monocytes were isolated from buffy coats of healthy donors (Sanquin) by Lymphoprep (Axis-Shield) gradient followed by Percoll (Amersham Biosciences) gradient steps. Monocytes were differentiated into immature DCs in the presence of 500 U/ml IL-4 and 800 U/ml GM-SCF (both Invitrogen) for 6–7 days in RPMI supplemented with 10% fetal calf serum, 10 U/ml penicillin, 10 mg/ml streptomycin (all Invitrogen) and 2 mM L-glutamine (Lonza). This study was done in accordance with the ethical guidelines of the Academic Medical Center.

Dermal DCs (DDCs) were isolated from human skin tissue obtained from healthy donors after corrective breast or abdominal surgery. A dermatome (Zimmer) was used to produce 0.3 mm skin grafts that were treated with dispase (1U/ml, Roche) for 45 min at 37°C to separate dermis and epidermis. Dermal tissue was floated on medium for 16h. Migrated cells were collected and separated based on CD14 (130-050-201, Miltenyi) and CD1c (130-090-506, Miltenyi) expression using magnetic beads according to the manufactures instruction. Isolated cells were analyzed for HLA-DR-PE/Cy7 (1:200, 560651, BD), CD11c-Alexa647 (1:100, 2108100, BioLegend), CD14-PerCP (1:10, 345786 BD) and CD1c-APC/Cy7 (1:50, 331519, BioLegend) expression on a BD Canto II (S6 Fig). CD14^+^ DDCs were characterized as HLA-DR^+^CD11c^+^CD14^+^CD1c^+^ and CD1c^+^ DDCs as HLA-DR^+^CD11c^+^CD1c^+^CD14^-^. Purity of sorted cells was over 95%.

DCs were stimulated with 1 μg/ml poly(I:C)LyoVec LMW or 10 μg/ml 5’ppp-dsRNA-LyoVec (both Invivogen) unless stated otherwise. DENV replication inhibitor SDM25N (10μM, Tocris Bioscience),blocking IFNα/βR antibody or blocking IFNLR antibody (20 μg/ml, both PBL Interferon Source) were added simultaneous with DENV to DCs.

DCs were transfected with 500 nM short interfering RNAs (siRNAs) using the Neon Transfection System (ThermoFisher) according to the manufacturer’s instructions. In brief, DCs were washed with PBS, resuspended in Buffer R (ThermoFisher) and divided over different siRNAs. DCs were transfected with a single pulse of 1500V for 20 ms, mixed with complete RMPI and incubated for 48h before stimulation. SMARTpool siRNA used were MAVS (M-024237-02), TRIF (M-012833-02), MYD88 (M-004769-01), RIG-I (M-012511-01), MDA5 (M-013041-00), TBK1 (M-003788-02), IKKε (M-003723-02), IRF9 (M-020858-02), IRF3 (M-006875-02), and non-targeting siRNA (D-001206-13) as control (all Dharmacon). Silencing was confirmed by real-time PCR, flow cytometry and immunoblot (S3 Fig).

### T cell differentiation and B cell activation

Naive CD4^+^ T cells were isolated from buffy coats of healthy blood donors (Sanquin) with human CD4^+^ T-cell isolation kit II (Miltenyi) by negative selection and subsequent depletion of CD45RO^+^ memory T cells using phycoerythrin (PE)-conjugated anti-CD45RO (80μg ml^-1^; R0843; Dako) and anti-PE beads (Miltenyi). B cells were isolated from buffy coats of healthy blood donors (Sanquin) with human B-cell isolation kit II (Miltenyi) by negative selection. This study was approved by the Medical Ethics Review Committee of the AMC.

DCs were either silenced for indicated proteins or treated with SDM25N and stimulated for 48h as indicated. DCs were combined with allogeneic naïve CD4^+^ T cells (5,000 DCs/20,000 T cells) in the presence of 10 pg/ml *Staphylococcus aureus* enterotoxin B (Sigma). SDM25N (1 μM, Tocris Bioscience) was added to cocultures of SDM25N-treated DCs to maintain inhibition of DENV replication. Neutralizing antibodies against IL-27 (5 μg/ml, AF2526; R&D Systems) or normal goat IgG (AB-108-C; R&D Systems) as isotype control was added at the start of DC-T cell coculture. After 3 days, cells were further cultured in the presence of 10 U/ml IL-2 (Chiron). Resting T cells were restimulated with 100 ng/ml PMA and 1 μg/ml ionomycin (both Sigma) for 24h. For flow cytometry analysis of restimulated T cells, cells were stained with Alexa Fluor 647-conjugated anti-CXCR5 (1:800; 558113; BD Pharmingen) and PerCP-Cy5.5-conjugated α-PD-1 (1:50; 561273; BD) before fixation in 2% *para*-formaldehyde for 20 min, followed by permeabilization in 50% methanol at -20°C for 45 min. Cells were stained with anti-Bcl-6 (1:50; ab19011; Abcam), followed by incubation with PE-conjugated anti-rabbit (1:200; 711-116-152, Jackson ImmunoResearch). Cells were analyzed on a FACS Canto II (BD Biosciences). Supernatants of restimulated T cells were harvested after 24h and IL-21 expression was analyzed by ELISA (eBioscience).

T-cell dependent B-cell activation was assessed by coculturing resting differentiated T cells restimulated with 1 μg/ml anti-CD3 (1XE, Sanquin) and 2 μg/ml anti-CD28 (15E8, Sanquin) with allogeneic B cells (100,000 T cells/50,000 B cells). Supernatants were harvested after 7 days for analysis of IgM and IgG production by ELISA (eBioscience).

### Virus production and infection

DENV-2/16681 was added to 80% confluent C6/36 cells at an MOI of 0.01 in RPMI medium RPMI supplemented with 2% fetal calf serum, 10 U/ml penicillin, 10 mg/ml streptomycin (all Invitrogen) and 2 mM L-glutamine (Lonza). After 5–7 days, supernatant was harvested and cleared from cellular debris by centrifugation and subsequent filtration using a 0.2 μM filter. Supernatant was aliquoted, snap-frozen in liquid nitrogen and stored at -80°C. Viral titers were determined as described previously[57].

DCs were infected with DENV at an MOI of 1 unless stated otherwise. Infection was determined after 36-48h by flow cytometry. Cells were fixed in 4% *para*-formaldehyde for 15 min followed by permeabilization in PBS supplemented with 0.1% saponin for 10 min. Cells were stained with anti-NS3 (1:800, SAB2700181, Sigma) followed by PE-conjugated anti-rabbit (1:200; 711-116-152, Jackson ImmunoResearch) in combination with APC-conjugated CD83 (1:25, 551073, BD Pharmingen) and FITC-conjugated CD86 (1:25, 555657, BD Pharmingen). Cells were analyzed on a FACS Canto II (BD Biosciences).

### Protein phosphorylation

TBK1, IKKε and STAT1 phosphorylation was determined by flow cytometry and immunoblot. For flow cytometry, cells were fixed in 4% *para*-formaldehyde for 15 min followed by permeabilization in 90% methanol at -20°C for 45 min. Cells were stained with phospho-specific antibodies against TBK1 Ser172 (1:50, 5483S, Cell Signaling), IKKε Ser172 (1:50, 06–1340, Millipore), STAT1 Ser708 (1:100, provided by M. Gale, Jr, University of Washington School of Medicine, Seattle, WA, (Perwitasari et al., 2011) or STAT1 Ser727 (1:200, 9177; Cell Signaling), followed by PE-conjugated anti-rabbit (1:200; 711-116-152, Jackson ImmunoResearch). Cells were analyzed on a FACS Calibur (BD Biosciences).

For immunoblot, whole cell extracts were prepared using Ripa lysis buffer (Cell Signaling Technology) and protein were resolved by SDS-PAGE and detected with anti-TBK1 Ser172 (1:1000, 5483S, Cell Signaling) anti-IKKε Ser172 (1:1000, 06–1340, Millipore), anti-STAT1 Ser708 (1:1000, provided by M. Gale, Jr, University of Washington School of Medicine, Seattle, WA, (Perwitasari et al., 2011) or anti-STAT1 Ser727 (1:1000, 9177; Cell Signaling). Membranes were also probed with anti-TBK1 (1:1000, 3504, Cell Signaling), anti-IKKε (1:1000, 2905, Cell Signaling) or anti-STAT1 (1:1000, 9172, Cell Signaling) as loading control. Primary antibody was detected using HRP-conjugated secondary antibody (1:2000, 21230, Pierce)

### Real-time quantitative PCR

mRNA was isolated using mRNA capture kit (Roche) and cDNA was synthesized with reverse transcriptase kit (Promega). PCR amplification was performed in the presence of SYBR Green in an ABI 7500 Fast PCR detection system (Applied Biosystems). Specific primers were designed using Primer Express 2.0 (Applied Biosystems; S1 Table). Expression of target genes was normalized to GAPDH (*N*_t_ = 2^Ct(GAPDH)–Ct(target)^) and set at 1 in DENV-infected DCs for each donor within one experiment.

### Cellular location of IRF3 and IRF9

Nuclear translocation of IRF3 and IRF9 was determined by confocal microscopy, immunoblot and ELISA. For Confocal microscopy, cells were allowed to adhere to poly-l-lysine coated glass slides for 20 min at 37°C before fixation in 2% *para*-formaldehyde for 20 min followed by permeabilization using 0.2% Triton for 10 min. Cells were stained with anti-IRF3 (1:100, D83B9, Cell Signaling) or anti-IRF9 (5 μg/ml, sc-496X, Santa Cruz) and anti-DENV E protein (1:400, 3H5-1, Millipore) followed by Alexa Fluor 488-conjugated anti-mouse (1:400, A11029, Invitrogen) and Alexa Fluor 546-conjugated anti-rabbit (1:400, A11035, Invitrogen). Nuclei were stained using Hoechst (1:10,000, Molecular Probes). Cells were analyzed on a Leica TCS SP8 X mounted on a Leica DMI6000 inverted microscope and data was processed using Leica LAS-X software.

For immunoblot, nuclear and cytoplasmic extracts were prepared using NucBuster protein extraction kit (Novagen). Proteins were resolved by SDS-PAGE and detected by immunoblotting with anti-iRF3 (1:1000, 4302; Cell Signaling) or anti-IRF9 (1:1000, sc-496, Santa Cruz). Membranes were also probed with anti-β-actin (1:2500, sc-81178; Santa Cruz) to ensure equal protein loading. Detection was performed as described above. Specific increase of IRF3 and IRF9 in the nuclear fraction without increase in the cytoplasmic fraction underscores specificity of the fractionation. IRF3 and IRF9 levels in nuclear extracts was also determined using ELISA (IRF3, SEB589HU, USCN Life Sciences; IRF9, MBS921012, MyBiosource).

### Statistical analysis

Statistical analyses were performed using the Student’s *t-*test for paired observations. Statistical significance was set at *P*<0.05.

## Supporting information

S1 Fig DENV efficiently infects DCs and induces DC maturation. (A,B) Flow cytometry analysis of DENV NS3 expression in DCs infected with DENV at an MOI of 10 (A) or indicates MOI (B) for 48h. Number above gate indicates percentage of gated cells. (C) Flow cytometry analysis of CD83 and CD86 expression on DENV-infected DCs after 48h. Data are representative of at least six (C) or two (A,B) independent experiments (mean ± s.d. of duplicates in B) with different donors.
(PDF)

S2 Fig DENV induced ISG expression depends on DENV RNA replication, MAVS, RIG-I and MDA5. (A,C,D) mRNA analysis of DENV (A), MxA, APOBEC3G and ADAR1 (C,D) RNA expression in mock-treated or DENV-infected DCs 24h post infection in the presence or absence of SDM25N (A) or after MAVS, TRIF, MYD88 (C), RIG-I or MDA5 (D) silencing by RNA interference using real-time PCR. Results were normalized to GAPDH and set at 1 in DENV-infected samples treated with DMSO. (B) DCs were treated and infected similarly as in (A) but DENV NS3 expression was measured 48h post infection using flow cytometry. Data are collated (mean ± s.d.) of at least three (A-D) different donors. **P<0.01 (student’s t-test).
(PDF)

S3 Fig Silencing efficiency of RIG-I, MDA5, MAVS, TRIF, MYD88, TBK1, IKKε, IRF3, IRF9. (A) Silencing of indicated proteins using RNA interference was confirmed by real-time PCR. mRNA expression was normalized to GAPDH and set at 1 in cells treated with control siRNA. (B,C) Silencing of indicated proteins using RNA interference was confirmed by flow cytometry (B) or immunoblot (C). Data in (A) are collated (mean ± s.d.) of at least eleven (RIG-I, MDA5), nine (MAVS, MYD88), five (TRIF, TBK1, IKKε, IRF3), or two (IRF9) independent experiments with different donors. Data in (B,C) are representative of at least two independent experiments with different donors.
(TIF)

S4 Fig Functional silencing of MAVS, TRIF and MYD88. (A,B,C) mRNA expression of IFN-β in untreated, RLR ligand poly(I:C)LyoVec (A), TLR3 ligand poly(I:C) (B) or TLR7/8 ligand R848 (C) stimulated DCs after silencing of MAVS (A), TRIF (B), or MYD88 (C) was determined by real-time PCR. mRNA expression was normalized to GAPDH and set at 1 for control-silenced cells. Data in (A-C) are collated (mean ± s.d.) of two different donors. *P<0.05, **P<0.01 (student’s t-test). -, unstimulated, p(I:C)LV, poly(I:C)LyoVec.
(PDF)

S5 Fig RLR triggering by poly(I:C)Lyovec or DENV does not lead to IL-12p70 or Activin A, while DENV RNA replication induces IL-27. (A) DCs were stimulated with poly(I:C)Lyovec, LPS, DENV MOI 1 or left untreated and mRNA expression of indicated genes was determined using real time PCR. mRNA expression was normalized to GAPDH and set at 1 for LPS-stimulated cells. Data are collated (mean ± s.d.) of three different donors. (B) Similar as in (A) but cell culture supernatant was analyzed for IL-12p70 by ELISA. Data show mean ± s.d. of duplicates and are representative of two different experiments with different donors. (C) mRNA analysis of IL-27p28 or IL-27 EBI3 expression in mock-treated or DENV infected DCs 24h post infection in the presence or absence of SDM25N using real-time PCR. Results were normalized to GAPDH and set at 1 in DENV infected samples treated with DMSO. Data are collated (mean ± s.d.) of four (C) or three (A) different donors or are representative of two (B) different experiments with different donors (mean ± s.d. of duplicates in C) *P<0.05, **P<0.01 (student’s t-test). -, unstimulated; p(I:C)LV, poly(I:C)Lyovec.
(PDF)

S6 Fig Flow cytometry analysis of human dermal DCs. Flow cytometry analysis of migrated cells from human dermal sheets after 16h. CD14^+^ DDCs were characterized as HLA-DR^+^CD11c^+^CD14^+^CD1c^+^ and CD1c^+^ DDCs as HLA-DR^+^CD11c^+^CD1c^+^CD14^-^.
(PDF)

S1 Table RT-qPCR primer sequences used for mRNA analysis. (PDF)
