# Supplementary figures and images for: RIG-I-like receptor activation by dengue virus drives follicular T helper cell formation and antibody production
Source: PLoS Pathog. 2017 Nov 29;13(11):e1006738. doi: 10.1371/journal.ppat.1006738 (PMC5724900; doi:10.1371/journal.ppat.1006738)

A

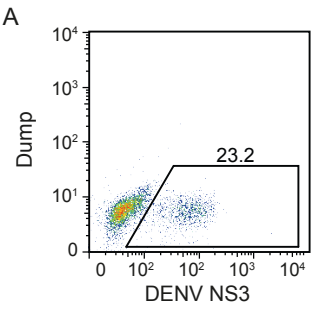

B

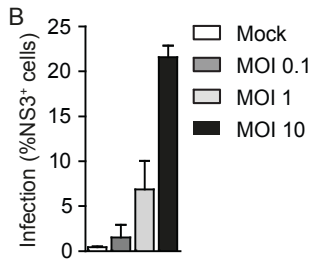

C

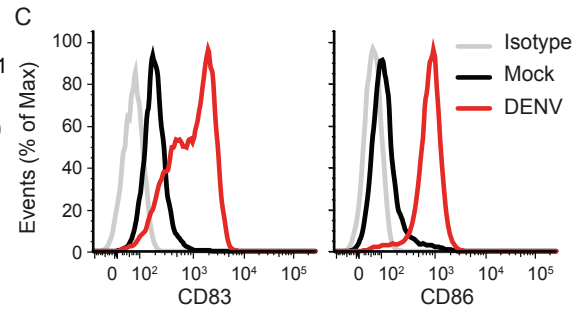

Supplement: S1 Fig — (A,B) Flow cytometry analysis of DENV NS3 expression in DCs infected with DENV at an MOI of 10 (A) or indicates MOI (B) for 48h. Number above gate indicates percentage of gated cells. (C) Flow cytometry analysis of CD83 and CD86 expression on DENV-infected DCs after 48h. Data are representative of at least six (C) or two (A,B) independent experiments (mean ± s.d. of duplicates in B) with different donors. (PDF) [file ppat.1006738.s001.pdf]

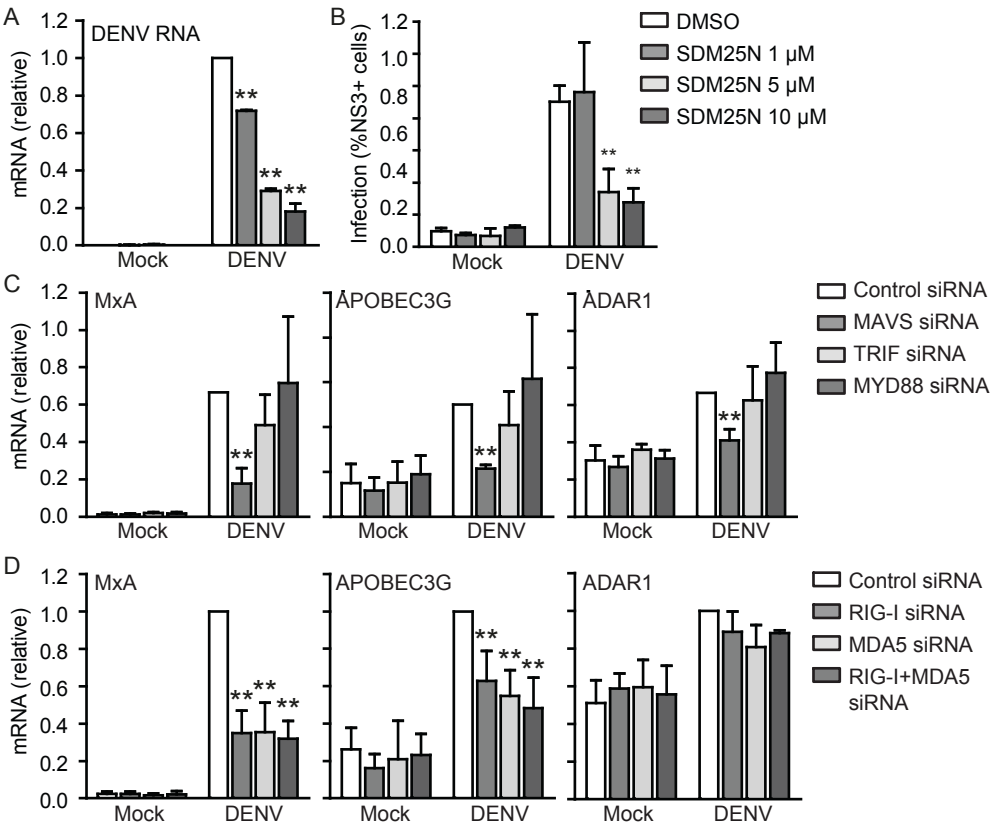

Supplement: S2 Fig — (A,C,D) mRNA analysis of DENV (A), MxA, APOBEC3G and ADAR1 (C,D) RNA expression in mock-treated or DENV-infected DCs 24h post infection in the presence or absence of SDM25N (A) or after MAVS, TRIF, MYD88 (C), RIG-I or MDA5 (D) silencing by RNA interference using real-time PCR. Results were normalized to GAPDH and set at 1 in DENV-infected samples treated with DMSO. (B) DCs were treated and infected similarly as in (A) but DENV NS3 expression was measured 48h post infection using flow cytometry. Data are collated (mean ± s.d.) of at least three (A-D) different donors. **P<0.01 (student’s t-test). (PDF) [file ppat.1006738.s002.pdf]

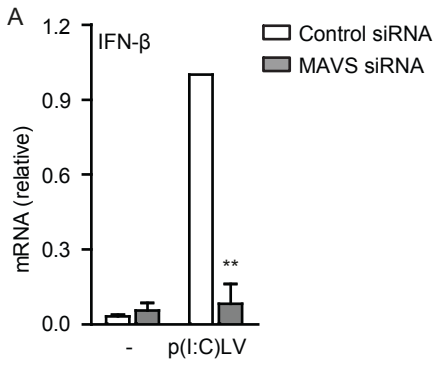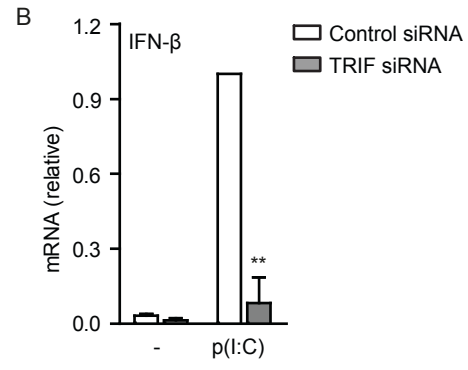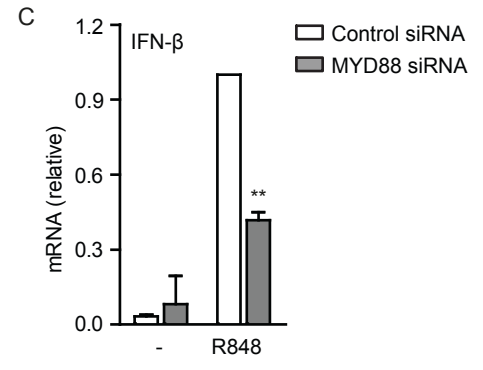

Supplement: S4 Fig — (A,B,C) mRNA expression of IFN-β in untreated, RLR ligand poly(I:C)LyoVec (A), TLR3 ligand poly(I:C) (B) or TLR7/8 ligand R848 (C) stimulated DCs after silencing of MAVS (A), TRIF (B), or MYD88 (C) was determined by real-time PCR. mRNA expression was normalized to GAPDH and set at 1 for control-silenced cells. Data in (A-C) are collated (mean ± s.d.) of two different donors. *P<0.05, **P<0.01 (student’s t-test). -, unstimulated, p(I:C)LV, poly(I:C)LyoVec. (PDF) [file ppat.1006738.s004.pdf]

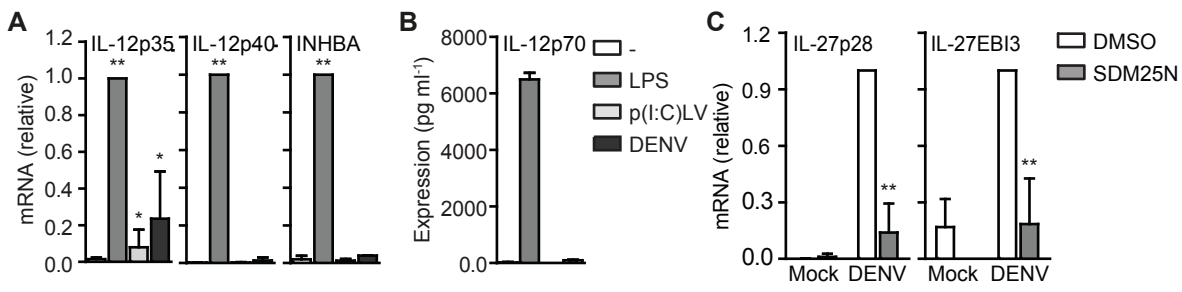

Supplement: S5 Fig — (A) DCs were stimulated with poly(I:C)Lyovec, LPS, DENV MOI 1 or left untreated and mRNA expression of indicated genes was determined using real time PCR. mRNA expression was normalized to GAPDH and set at 1 for LPS-stimulated cells. Data are collated (mean ± s.d.) of three different donors. (B) Similar as in (A) but cell culture supernatant was analyzed for IL-12p70 by ELISA. Data show mean ± s.d. of duplicates and are representative of two different experiments with different donors. (C) mRNA analysis of IL-27p28 or IL-27 EBI3 expression in mock-treated or DENV infected DCs 24h post infection in the presence or absence of SDM25N using real-time PCR. Results were normalized to GAPDH and set at 1 in DENV infected samples treated with DMSO. Data are collated (mean ± s.d.) of four (C) or three (A) different donors or are representative of two (B) different experiments with different donors (mean ± s.d. of duplicates in C) *P<0.05, **P<0.01 (student’s t-test). -, unstimulated; p(I:C)LV, poly(I:C)Lyovec. (PDF) [file ppat.1006738.s005.pdf]

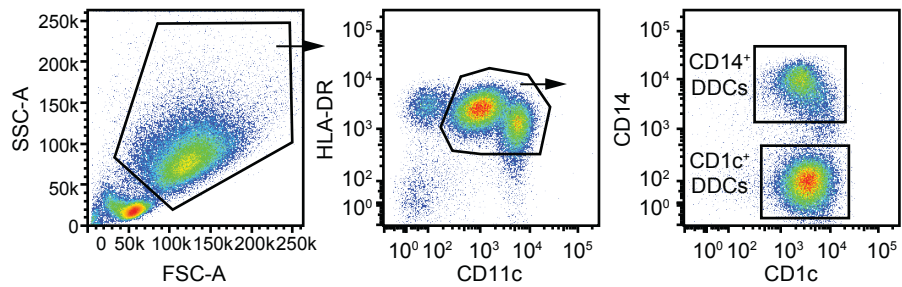

Supplement: S6 Fig — Flow cytometry analysis of migrated cells from human dermal sheets after 16h. CD14+ DDCs were characterized as HLA-DR+CD11c+CD14+CD1c+ and CD1c+ DDCs as HLA-DR+CD11c+CD1c+CD14-. (PDF) [file ppat.1006738.s006.pdf]
