# Supplementary material for: RIG-I-like receptor activation by dengue virus drives follicular T helper cell formation and antibody production
Source: PLoS Pathog. 2017 Nov 29;13(11):e1006738. doi: 10.1371/journal.ppat.1006738 (PMC5724900; doi:10.1371/journal.ppat.1006738)
Supplement: S1 Table — (PDF) [file ppat.1006738.s007.pdf]

Gene product	Forward primer	Reverse primer
IFN-α	GACTCCATCTTGGCTGTGA	TGATTTCTGCTCTGACAACCT
IFN-β	ACAGACTTACAGGTTACCTCCGAAAC	CATCTGCTGGTTGAAGAATGCTT
MxA	TTCAGCACCTGATGGCCTATC	GTACGTCTGGAGCATGAAGAACTG
APOBEC3G	TTGAGCCTTGGAATAATCTGCC	TCGAGTGTCTGAGAATCTCCCC
ADAR1	ATCAGAACCCCCACTCACCC	TGCCTCGCTCTCTTCCTACAAC
TRIM5α	AGAACATACGGCCTAATCGGC	CAACTTGACCTCCCTGAGCTTC
IL-27p28	GCTTTGCGGAATCTCACCTG	TGAAGCGTGGTGGAGATGAAG
IL-27EBI3	GGCTCCCTACGTGCTCAATG	GGGTCGGGCTTGATGATGT
RIG-I	CCAAGCCAAAGCAGTTTTCAAG	CATGGATTCCCCAGTCATGG
MDA5	TGAGAGCCCTGTGGACAACC	CGCTGCCCACTTAGAGAAGC
MAVS	TGATTTCCTCGCAATCAGACG	GAAGCCGATTTCCAGCTGTATG
TRIF	CTGGAGCCGGTCAAAAACC	ACGGCTTGGTATTTGGAGAGG
MYD88	GTGTCTGACCGCGATGTCC	ACAACCACCACCATCCGG
TBK1	TTACAGGAAAGCCTTCTGGTGC	TCCACTCCAGTCAATTGGTCC
IKKε	TTGGAGTGACCTTGTACCATGC	CATGATCTCCTTGTTCCGCC
IRF3	AAGGAGGCGTGTTTGACCTG	CATAGCGTGGTGAGCGT
IRF7	GCTCCCCACGCTATACCATCTAC	GCCAGGGTTCCAGCTTCAC
IRF9	ACACTCAGTCCCTCTGTGCTCC	CTGAATGGACTGCTCCCCC
GAPDH	CCATGTTCGTCATGGGTGTG	GGTGCTAAGCAGTTGGTGGTG
IFNL1	GAGGCCCCCAAAAAGGAGTC	AGGTTCCCATCGGCCACATA
IFNL2	AGGCCCCAAAAAAGGAGTCC	CAACACAATTCAGGTCTCGCG
IFNL3	AGGCCCCAAAAAAGGAGTCC	CAACACAATTCAGGTCTCGCG
IFNL4	ATCGCATTGGTTGTTTTCCG	AAACTCAGGCCACCTGAGTCC
INHBA	ACGGGTATGTGGAGATAGAGG	TGGAAATCTCGAAGTGCAGC
DENV-2 RNA	AAGGTGAGATGAAGCTGTAGTCTC	CATTCCATTTTCTGGCGTTCT
